# Laser Texturing as a Way of Influencing the Micromechanical and Biological Properties of the Poly(L-Lactide) Surface

**DOI:** 10.3390/ma13173786

**Published:** 2020-08-27

**Authors:** Magdalena Tomanik, Magdalena Kobielarz, Jarosław Filipiak, Maria Szymonowicz, Agnieszka Rusak, Katarzyna Mroczkowska, Arkadiusz Antończak, Celina Pezowicz

**Affiliations:** 1 Department of Mechanics, Materials and Biomedical Engineering, Wrocław University of Science and Technology, 50-370 Wrocław, Poland; magdalena.tomanik@pwr.edu.pl (M.T.); jaroslaw.filipiak@pwr.edu.pl (J.F.); celina.pezowicz@pwr.edu.pl (C.P.); 2 Department of Experimental Surgery and Biomaterials Research, Faculty of Dentistry, Wroclaw Medical University, 50-326 Wrocław, Poland; maria.szymonowicz@umed.wroc.pl; 3 Division of Histology and Embryology, Department of Human Morphology and Embryology, Faculty of Medicine, Wroclaw Medical University, 50-368 Wrocław, Poland; agnieszka.rusak@umed.wroc.pl; 4 Laser and Fiber Electronics Group, Faculty of Electronics, Wroclaw University of Science and Technology, 50-372 Wrocław, Poland; katarzyna.mroczkowska@pwr.edu.pl (K.M.); arkadiusz.antonczak@pwr.edu.pl (A.A.)

**Keywords:** poly(L-lactide), laser irradiation, surface enhancement, micromechanical properties, cytotoxicity

## Abstract

Laser-based technologies are extensively used for polymer surface patterning and/or texturing. Different micro- and nanostructures can be obtained thanks to a wide range of laser types and beam parameters. Cell behavior on various types of materials is an extensively investigated phenomenon in biomedical applications. Polymer topography such as height, diameter, and spacing of the patterning will cause different cell responses, which can also vary depending on the utilized cell types. Structurization can highly improve the biological performance of the material without any need for chemical modification. The aim of the study was to evaluate the effect of CO_2_ laser irradiation of poly(L-lactide) (PLLA) thin films on the surface microhardness, roughness, wettability, and cytocompatibility. The conducted testing showed that CO_2_ laser texturing of PLLA provides the ability to adjust the structural and physical properties of the PLLA surface to the requirements of the cells despite significant changes in the mechanical properties of the laser-treated surface polymer.

## 1. Introduction

Poly(L-lactide) (PLLA) is one of the more technologically advanced polymers. High product purity and high polymer biocompatibility are especially important for medical applications. Polymeric materials based on PLLA are often used for the production of, among others, bioresorbable screws, surgical sutures, vascular stents, and bone scaffolds [1,2,3]. Some medical devices, such as bone scaffolds, are complex structures of small geometrical dimensions, whose shaping by conventional methods, e.g., by injection or mechanical micromachining, is technologically challenging. The assumptions made at the design stage concerning the accuracy of execution of small-scale components cannot always be achieved using conventional processing methods.

The interaction of cells with the surfaces of biomaterials is a complex phenomenon that depends on many factors. The properties of an implant surface, including roughness, topography, and wettability directly affect tissue cells and must be adjusted to their mechanobiology [4,5,6]. The hydrophobic nature of PLLA [7,8,9] and its low surface roughness [10] adversely affect the adhesion of cells to the surface of the material [11], hence it is necessary to modify its surface properties. One of the possible ways of adjusting the material to cell preferences is to modify only its topography [4,5,12,13] without the need to interfere in the chemical bulk composition of the polymer. Technologies based on material processing with a laser beam are an excellent solution, enabling selective surface structuring of even very geometrically complex objects, regardless of their dimensions [12,14,15]. One way to structure the surfaces of biodegradable polymers is to irradiate the materials with a CO_2_ laser beam [16,17,18,19,20,21]. It is known that a CO_2_ laser surface irradiation causes formation of nano- and microstructures on the surfaces of the treated materials [10,21] which can alter their mechanical characteristics [21,22]. However, the use of a laser treatment process, due to the associated thermal effects, may adversely affect the material and change its properties [16,21,23,24,25,26,27]. The physicochemical properties of the irradiated material depend on the type of laser source and parameters of the irradiation process, such as, for example, the light wavelength, pulse duration, and number and energy of pulses.

A reliable assessment of the impact of laser irradiation on the degree of polymer degradation requires an analysis of a wide range of process parameters used during the processing of this material. In our previous work [16,27], an analysis of the influence of CO_2_ laser energy density on the degree of PLLA degradation was initially conducted in the fluence range from 13 to 95 J/cm^2^, i.e., from the minimum value that could be set on our laser system, to the value that ablated the material about half its thickness (for films with a thickness of about 250 µm). The analysis of all changes in the physicochemical properties was subordinated to the overriding goal of micro-processing of these polymers. The aim was to obtain information on how this polymer degrades (and what consequences it has) during the production of practically usable implants, i.e., stents [15,16,27]. Performing further research for such a large number of cases/fluences would be redundant, as certain regularities were observed for specific ranges of fluence. Therefore, we limited our considerations to three characteristic surface fluences: 24, 48, and 71 J/cm^2^ [21,22]. The first fluence (24 J/cm^2^) is the maximum value of the energy density at which neither surface layer melting nor ablation was observed. Despite this, significant changes were observed in both differential scanning calorimetry (DSC) and, above all, gel permeation chromatography (GPC) [16,27] (bimodal distribution and significant loss of molecular weight in the near-surface layer). In this case, however, Fourier transform infrared reflectance (FTIR) spectroscopy showed no changes. The erasure of the ageing peak in the DSC thermograms indicated that the polymer had crossed the glass transition in all its volume. The second fluence (48 J/cm^2^) corresponded to the case of remelting the PLLA top layer. There were already visible changes (apart from those recorded for 24 J/cm^2^ in GPC and DSC) in FTIR spectroscopy (appearance of new, characteristic peaks of vinyl groups, ketones, etc.). This fluence could also be considered the beginning of the polymer ablation threshold. The third characteristic fluence (71 J/cm^2^) is the value at which intense ablation was noted. Here, the visible changes were similar but more intense compared to the values observed in GPC, FTIR, and DSC for 48 J/cm^2^ [16,27].

Therefore, the aim of the study was to evaluate the effect of irradiation of PLLA with a CO_2_ laser using different accumulated fluences, resulting in the formation of micro-groove patterns on the surface and affecting the micromechanical and cytotoxic properties of the material.

## 2. Material and Methods

### 2.1. Material and Surface Modification

The tests were carried out using PLLA (L210S, Evonik Industries AG, Essen, Germany), which underwent the process of formation described in detail in previous studies by our group [21,22]. Briefly, thin films of the material were produced by heating the granulate to a temperature of 200 °C, followed by melt processing. The specimens were excised from the obtained amorphous sheets with the crystallinity of *X_c_* ≈ 2% and average thickness of 350 μm. Biological testing was performed on discs with a diameter of 12 mm, while other tests used rectangular specimens measuring 20 × 5 mm.

All the specimens were irradiated using the Speedy 300 (Trotec Laser GmbH, Marchtrenk, Austria) engraving system, equipped with an air-cooled RF-excited (Radio Frequency-excited) pulsed CO_2_ laser (10.6 ± 0.03 µm) (Series 48-2, Synrad Inc. Mukilteo, WA, USA) with the maximum average power of 25 W and pulse duration ranging from several dozen to several hundred μs depending on the power and the pulse repetition rate. The beam with the mode purity TEM_00_ at 95% (beam quality factor M^2^ < 1.2) was focused on the surface of the material using a 38.1 mm (1.5”) focal length lens. The beam waist width was about 2w_0_ = 125 μm. The tests were conducted for the experimentally determined range of parameters (Table 1).

The specimens intended for cytotoxicity testing were subjected to plasma sterilization. The PLLA specimens were sterilized in a Sterrad 100S plasma sterilizer (Advanced Sterilization Products, Irvine, CA, USA) at a temperature of 46 °C, with 2 exposures to hydrogen peroxide lasting 7 min each, under general sterilization conditions lasting 51 min.

### 2.2. Roughness and Wettability

In order to evaluate the surface properties of the PLLA thin films, both referenced (Ref) and micro-patterned (F_1_–F_3_) surfaces were examined. Surface roughness was determined based on height profiles using a Micro Combi Tester (CSM Instruments SA, Peseux, Switzerland) in the scratch test mode with a Rockwell indenter (CSM Instruments SA, Peseux, Switzerland) 10 µm in diameter. Unlike traditional profilometers, the optical module of the device with a magnification of the order of 50–200 times allowed for precise selection of the profile location and correlation of the profile curves with the recorded surface geometry of the specimens. One of the advanced scratch test (pre-scan) settings allowed us to register a profile so that after performing the proper scratch test it was possible to determine the actual penetration depth of the indenter into the material. This option was used to register surface profiles of the analyzed specimens. The contact force of the intender was set to 10 mN, so no scratches were observed on the surface of the material after the profile was made. The methodology used to determine the surface roughness was validated on surface roughness reference specimens with R_z_ = 9.47 µm, achieving a compliance level of 98%.

Six profile measurements were performed on the specimens from each measurement group in the direction perpendicular to the direction of laser beam propagation over a measuring length of 1.25 mm. The profiles obtained from a pre-scan allowed us to determine the R_z_ parameter understood as height of the roughness profile for the highest 5 and the lowest 5 points on the specimens. Based on the above, the theoretical R*_a_* value (arithmetic mean of profile deviation) was calculated as 1/5 of the R_z_ value. Measurements were based on the ISO 4287 standard [28].

The effect of the developing roughness on the affinity of surfaces to the liquid(s) was examined by determining the wetting angle θ. Six drops with an average volume of 0.3 µl were applied on the examined surfaces; the tests were carried out at room temperature equal to 22 ± 1 °C using a Surftens Universal instrument (OEG Gesellschaft für Optik, Elektronik & Gerätetechnik mbH, Frankfurt (Oder), Germany).

### 2.3. In Vitro Cytotoxicity

The cytocompatibility effect of irradiation of the PLLA surface with a CO_2_ laser using different accumulated fluences was evaluated on a culture Balb/3T3 of normal mouse fibroblast cell line (clone A31, American Type Culture Collection (ATCC CCL-163), Manassas, VA, USA). The cells were cultured in Dulbecco’s Modified Eagle Medium (DMEM) with 4.5 g/L of glucose, 25 mM HEPES (Corning Inc., Corning, NY, USA), 1% L-glutamine with streptomycin and penicillin (Sigma-Aldrich, St. Louis, MO, USA), and 10% fetal bovine serum (Sigma-Aldrich, Saint Louis, Missouri, USA) under standard conditions of 37 °C and 5% CO_2_, at constant humidity. Balb/3T3 fibroblasts were trypsinized (0.25% Trypsin-EDTA, Sigma-Aldrich, Saint Louis, Missouri, USA), suspended in culture medium, and seeded on a 6-well plate (Falcon, Corning Inc., Corning, NY, USA) at a density of 1.5 × 10^5^ cells/well. After 24 h of cell incubation, discs of laser-treated and reference test material were applied to each well in such a way that the fibroblast monolayer was in contact with the polymer surface. The control group was cells without contact with the material. After 24 h, fibroblast morphology was assessed under the surface of the test material and at the edges of the specimens using a CKX-41 inverted phase contrast microscope (Olympus, Tokyo, Japan). To assess the cytotoxic effect [29], a grading scale was used where the changes in the culture over grade 2 (mild grade) were considered to be a cytotoxic effect [29]. On the other hand, a moderate degree of cytotoxicity was characterized by the presence of a zone of altered (degenerated or malformed) cells, which occurred only under the surface of the test material, with normal morphology of the cells around the material. Tests were also carried out on colonization of the test material by Balb/3T3 cells. Polymer specimens in the form of discs were placed in the wells of a 6-well plate (Falcon) and fibroblasts from the same culture were seeded on the polymer surface at a density of 1.5 × 10^5^ cells/well. After 24 h, cell morphology and adhesion to the surfaces of the test materials were assessed using a CKX-41 inverted phase contrast microscope.

### 2.4. Micromechanical Properties

Tests of micromechanical properties were carried out on a Micro Combi Tester (CSM Instruments SA, Peseux, Switzerland) using a Vickers prismatic indenter with an apex angle of 136°. Specimens with an average thickness of 350 μm were mounted on an aluminum test rig of much higher stiffness using a very thin layer of crystal bond glue. The table was immobilized with adjustable clamps of the microhardness tester. An indentation load of 100 mN was used based on pre-tests, which allowed us both to avoid the effect of substrate on the specimen and enabled testing of the entire thickness of the surface layer. The resulting indentation depths were in the range of 3–4 μm to 10–12 μm for all the specimens. The measurement points, i.e., 20 indentations for a particular irradiated type of surface, were selected in the visual mode enabling a preview of the polymer surface under an optical microscope.

The tests for each indentation were run at a constant strain rate during the loading and unloading phases. As the indenter tip approached a maximum depth at 100 mN, the test was paused for 10 s and then the specimen was unloaded at the same strain rate. The unloading section of these data were analyzed to calculate the mechanical properties using the Oliver Pharr model [30]. Poisson’s ratio (ν) of PLLA was set to 0.35, which is a representative value for polymers, while the parameters used for the diamond were E = 1140 GPa and ν = 0.07. The analysis focused on microhardness (H_IT_, HV), Young’s modulus (E_IT_), maximum indentation depth (hm) as well as plastic strain energy (Wp) and elastic strain energy (We).

### 2.5. Statistical Analysis

The obtained results of the micromechanical properties and wetting angle were subjected to statistical analysis, starting with checking the normality of the obtained distributions (Shapiro–Wilk test), and were tested for homogeneity of variance (Levene’s test). The results obtained within the study groups were averaged and standard deviations were determined. The statistical significance between individual groups was evaluated using one-way analysis of variance (one-way ANOVA) and post-hoc Tukey’s tests; the statistical analysis was carried out for a significance level of α = 0.05.

## 3. Results

### 3.1. Roughness and Wettability

**Figure 1:**
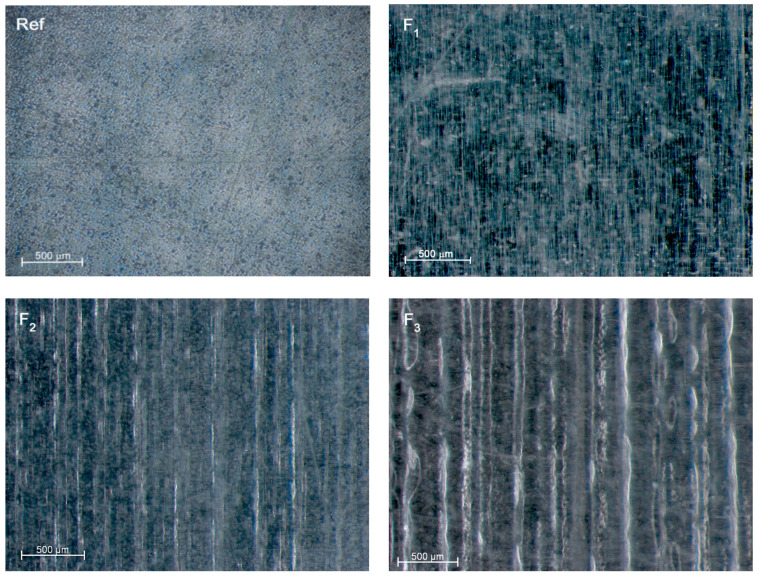
Microscopic images of the obtained surfaces (magnification 100×) recorded for reference material (Ref) and laser treated with accumulated fluences of 24 J/cm^2^ (F_1_), 48 J/cm^2^ (F_2_) and 71 J/cm^2^ (F_3_).

The microscopic evaluation of the irradiated specimens’ surfaces showed the presence of repetitive structures. As the laser irradiation parameters increased, the observed micro-patterns became larger (Figure 1).

The determined profiles of surface roughness showed that increased use of accumulated fluence created micro-grooves, making the surface increasingly rough. Table 2 shows averaged R_z_ values as well as R_a_ values determined on their basis.

**Figure 2:**
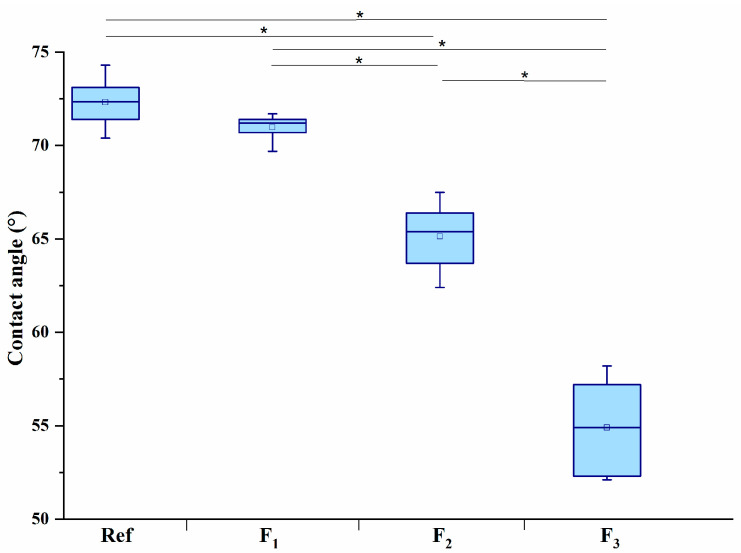
The wettability of the surface modified with different laser parameters; statistical significance between the study groups for water and PBS, obtained in one-way ANOVA and post-hoc Tukey’s tests; * *p* < 0.001.

The conducted tests showed that surface structuring with the use of a CO_2_ laser altered the wettability of poly(L-lactide) (Figure 2). The reference material was partially wettable, however, it was the most hydrophobic (θ = 72.3 ± 1.4°). Irradiation of the material with the lowest accumulated fluence (F_1_) was almost unnoticeable as a micro-pattern (Figure 1) and did not significantly affect the surface wettability. The use of a higher fluence caused a significant change in the nature of the surface, not only compared to the reference material but also between individual groups. Irradiation of the surface decreased the angle of wettability θ relative to the reference material by 9.7% and 24.1% for accumulated fluences of, respectively, 48 J/cm^2^ and 71 J/cm^2^, indicating that the surface became more hydrophilic. The smallest angle (θ = 54.9 ± 2.4°) was noted for an accumulated fluence of 71 J/cm^2^.

### 3.2. In Vitro Cytotoxicity

**Figure 3:**
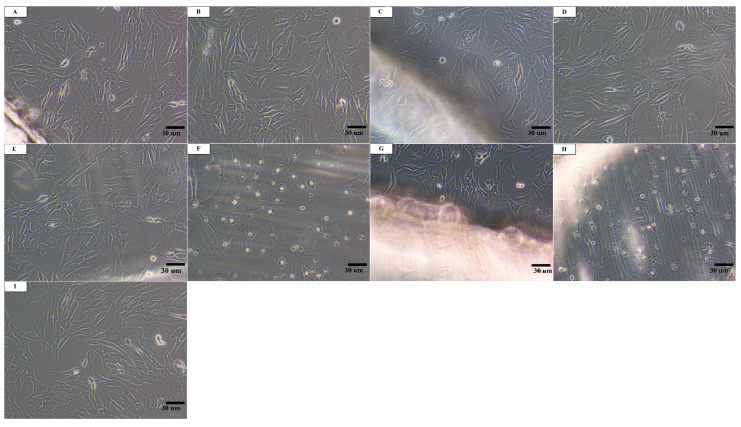
Culture of Balb/3T3 fibroblasts after 24-h contact with the test materials around and under the surface of the specimen: (**A**) and (**B**) Ref, (**C**) and (**D**) F_1_, (**E**) and (**F**) F_2_, (**G**) and (**H**) F_3_, and (**I**) control cell culture without contact with materials. Altered cells occur only in a limited zone under the specimens. Magnification: ×100.

Reference material and material after laser treatment with the fluence of F_1_ after application on fibroblast culture did not cause changes in the morphology of cells under the surface of the specimen, in the zone around the specimen, and in the zone outside of the specimen in the whole well, compared to the control cell culture (Figure 3). Materials after laser treatment F_2_ and F_3_ did not change the cell morphology compared to the control cell culture either in the zone around the laser-treated surface or in the zone outside of the specimen in the whole well. Changes in cell morphology, such as malformation or degeneration of cells, vacuolization, and also occasional lysis and growth inhibition were only observed for specimens that were laser treated with accumulated fluences F_2_ and F_3_ (Figure 3), which showed a mild degree (2) of cytotoxicity. According to the standard protocol stated in ISO 10993-5 for evaluation of in vitro cytotoxicity of medical devices, cytotoxicity effects can be considered when the reactivity grade of the cytotoxic effect is greater than 2 [29]. The conducted study showed that laser treatment changed the cytotoxicity of the materials against Balb/3T3 cells.

**Figure 4:**
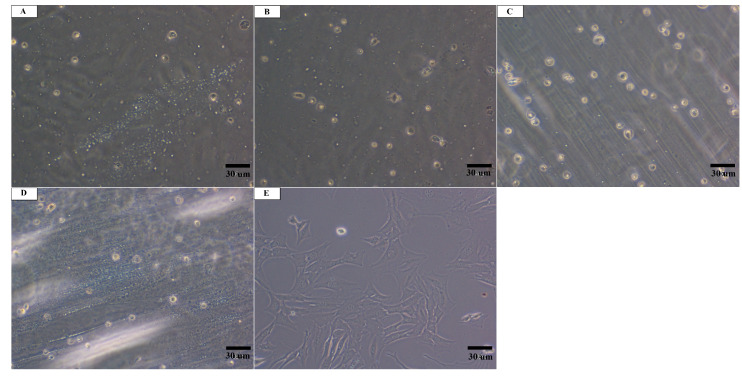
Culture of Balb/3T3 fibroblasts 24 h after application of the cells on the surfaces of the materials: (**A**) Ref, (**B**) F_1_, (**C**) F_2_, (**D**) F_3_, and (**E**) control cell culture without contact with the material No cell colonization of the material surfaces was observed. Magnification: ×100.

After 24 h after application of the culture, there was no observed cell adhesion to the surfaces of the test materials F_1_, F_2_, F_3_, and Ref (Figure 4). The surfaces of all test materials showed cells with a rounded phenotype, which did not coat the test specimen. The cells in the zone around the specimens and in the zone outside the specimens in the whole well showed normal morphology (Figure 4).

### 3.3. Micromechanical Properties

Micromechanical properties were examined during a typical instrumented indentation test with constant parameters over several repetitions for each type of specimens. The obtained parameters were characteristic for all types of irradiated materials and indicated a specific relation to accumulated fluences (Table 3).

**Figure 5:**
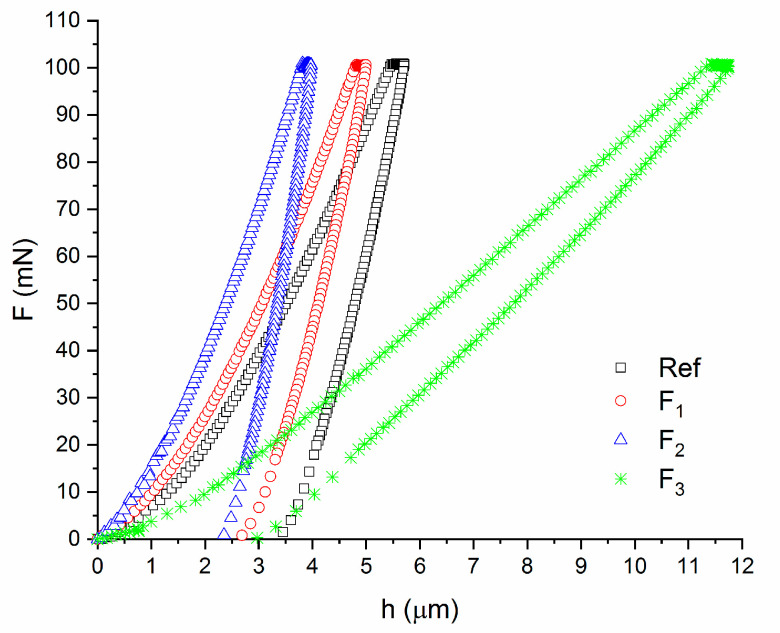
Typical loads vs. indentation depth curves for reference and irradiated PLLA specimens.

All mechanical parameters showed gradual changes with the trend maintained for all the obtained values, starting from the reference specimens to specimens irradiated with a medium accumulated fluence. However, at the fluence of 71 J/cm^2^, all cases showed rapid (abrupt) changes of the trend. Irradiation of PLLA with the lowest and medium fluencies affected the material surface by increasing its hardness and stiffness. Plastic and elastic strain energies gradually decreased. Resistance of the material from the side of the irradiated surface increased. The highest used fluence strongly changed the mechanical behavior of the PLLA specimen. The trends in changes of the mechanical parameters were significantly reversed (*p* < 0.05). The maximal depth during the testing was almost twice higher compared with reference specimens and three times higher compared with specimens irradiated with 48 J/cm^2^ fluence. PLLA became significantly more deformable and plastic than the reference material. This phenomenon is clearly visible in the relationships between the indentation force and depth (Figure 5). The mechanical curves obtained for the specimens irradiated with the fluences F_1_ and F_2_ were shifted as a result of indentation tests towards lower deformations with respect to the reference specimens while demonstrating the stiffer nature of the test surface. The relationships between the indentation force and depth for PLLA specimens irradiated with 71 J/cm^2^ were shifted in the opposite direction to all other curves.

## 4. Discussion

A series of tests focused on assessing changes in the biomaterial surface allowed us to determine the potential of CO_2_ laser texturing for biomedical applications. The properties of an implant surface, including roughness, topography, and wettability significantly determine the applicability of the implant and can be adjusted to the preferences of a given type of cell [4,5,6]. The almost completely smooth surface is not conducive to adhesion and proliferation of cells [10], requiring the use of additional material treatment. The biological potential of the surface can be increased by bulk modification of the material, mainly by altering the chemical composition [4,5,12,13] or by surface modification using, for example, a laser beam [10,12,14,15,31]. However, due to the action of the laser beam on the polymer material, photochemical degradation of the material may occur [24,25], significantly affecting the material properties of the polymer. Only a few studies have focused on assessing the impact of irradiation of biodegradable polymers with a CO_2_ laser on their mechanical [15,22] or physicochemical properties [16,32]. The use of a low value of accumulated fluence strengthens and stiffens the material, which is visible in uniaxial tensile tests [22] and under hydrolytic degradation [21]. On the other hand, the highest applied value of accumulated fluence makes the surface of the material more pliable, so it strongly deforms and plasticizes (Figure 1).

The results of wetting angle tests indicate that the reference PLLA surface is the most hydrophobic, which adversely affects cell adhesion to the surface of the material [11]. The obtained wetting angle values for the reference material are consistent with the data presented by other authors [7,8,9]. In the process of surface laser structuring, an increase in the used accumulated fluence was accompanied by a decrease in the contact angle, resulting in better wetting (wettability) of the material and an increased surface roughness. In contrast to other studies [16,33] showing no relationship between CO_2_ laser irradiation and wettability of the PLLA, our tests proved the significance of the process as there was an increase in hydrophilicity. A similar trend was also observed for other materials such as PEEK [34], LDPE [17] and nylon 6.6 [19], where CO_2_ laser also improved the wettability of those materials. Surface wettability, in general, is determined by the functional groups (chemical composition) present on the surface and by surface roughness [35]. The observed decrease in the contact angle of water wetting increasing surface hydrophilicity is partly attributed due to the larger surface roughness [35] and changing relationship between polar and nonpolar functional groups, as well as between acidic or basic sites available at the modified surface region [36]. The chemical structure of the laser-irradiated PLLA surface was demonstrated in our previous studies [16,22,27]. The Fourier transform infrared spectroscopy (FTIR) confirmed both a decrease in the number of aliphatic ester segments and the defragmentation of the main chain following C-C and C-H bond scission [37,38]. The ATR/FTIR also proved the appearance of a few small but characteristic absorption bands for vinyl groups (RCH=CH_2_), ketones (RCOCH=CH_2_), and/or vinyl ethers (ROCH=CH_2_) [22]. The appearance of these bands may indicate that the decomposition of PLLA by thermal CO_2_ laser impact occurred, among others, by means of the cis elimination reactions, in which double carbon bonds were formed in the vinyl groups (−CH=CH_2_) [16,22,27]. Admittedly, the use of X-ray photoelectron spectroscopy (XPS) did not demonstrate that processing with CO_2_ laser irradiation caused no oxidation of the surface layer, despite the presence of oxygen in the process environment. The value of O/C remained constant [27]. The lack of photo-oxidation may be due to the fact that the degradation process was initiated by temperature and such PLLA decomposition generally does not generate free radicals.

Cytotoxicity tests were performed after sterilization of the PLLA in low-temperature plasma called “cold” plasma. The plasma treatment is a surface process commonly used to clean (sterilize) or etch polymers, alter their surface wettability, and improve the cell affinity to the polymer surface, resulting in better cytocompatibility [36]. Surface wettability increases as a result of the formation of the new chemical groups, mainly polar, introduced to the polymer surface (regardless of the gas used for modification) [39]. The newly formed functional groups enhance cell adhesion [40], owing to chemical interaction and higher surface energy increasing adhesive strengths on the polymer surface [41]. Plasma treatment modifies the surface morphology [36]. However, low-temperature plasma modifies only the upper layer of the polymer surface [42], assessed as approximately 10 nm in depth or less in depth (R_a_ < 10 nm) [36]. Therefore, it is generally accepted that plasma discharge can modify polymer surfaces, whereas the bulk properties remain unchanged. On the other hand, sterilization with hydrogen peroxide gas plasma is the recommended procedure for heat-sensitive poly(lactic acid) [43]. Hence, the process of plasma treatment enhances surface wettability, while having a negligible effect on the mechanical properties of the PLLA surface, where the maximum indentation depth (hm) is measured in micrometers (Table 3). Plasma sterilization is a compromise between surface modification mainly improving cytocompatibility of PLLA surface and physicochemical modification of surface layers at a shallow depth. Please note that all the tests were performed on non-sterilized PLLA specimens except for cytotoxic tests.

Proliferation and differentiation of cells depend on many factors, including mechanical conditions. The optimal conditions for stable maintenance of the continuity of the life cycle and performance of the functions are different for each type of cell. Cells present in load-bearing tissues are sensitive to mechanical stimulations through mechanoreceptors and, accordingly, to load parameters such as amount, duration, or even amplitude. Consequently, different metabolic pathways are initiated. However, cytocompatibility of materials is viewed mainly in terms of surface topography and chemicals interacting with the cells, including products of material degradation. The influence of the mechanical properties of the materials that are colonized by the cells on cytocompatibility of the materials is negligent. According to previous research [19,44], osteoblasts choose surfaces with greater roughness and their division occurs much faster on smooth surfaces; moreover, increased surface roughness also provides them with better adhesion [10,17,19,34]. The use of laser treatment to significantly increase surface roughness allows an increase in proliferation and differentiation of bone-forming cells. The increase in roughness is associated with the formation of nano- [45] or micropatterns [21] on aliphatic polymer surfaces following laser irradiation. The irradiated PLLA surfaces showed parallel and equidistant grooves and ridges. The pattern repeated itself and as the fluence increased, so did the distance between each two adjacent ridges (profile element width). To the best of our knowledge, this is the first presentation of results of roughness of PLLA surfaces irradiated by a CO_2_ laser. Previously, R_a_ and R_z_ parameters were discussed mainly for PLLA combined with fillers in nano- and microscales, such as hydroxyapatite [46] or drug-intercalated fillers [47]. Roughness parameters of a mix of PLLA and particles (bulk modification) were comparable, or even higher, to those recorded here for PLLA irradiated with F_2_ and F_3_ fluences. Higher values of roughness parameters are justified if the bulk modification is processed by macromolecules incorporated into the polymer matrix and dependent on the concentration of the filler. Studies on commercially available implant materials, such as machined Ti6Al4V alloy, show R_a_ and R_z_ (0.48 ± 0.05 and 2.48 ± 0.29, respectively) strongly comparable to PLLA irradiated with 48 J/cm^2^ fluence [48]. However, better osteoblast proliferation was found in the materials (etched or plasma-sprayed titanium) with higher surface roughness (Ra > 3.5 μm) which is unattainable for laser processing.

In the research conducted by Zheng et al. [20], pre-osteoblast adhesion and proliferation increased after laser treatment. An enhancement in the biocompatibility of the CO_2_ laser-treated surfaces was also observed by Waugh et al. [19]. The conducted tests showed that surface modification with a CO_2_ laser wavelength affected the cell viability and improved cell growth. Moreover, cell coverage was greater on textured than on non-textured surfaces. As infrared spectroscopy revealed the presence of a vinyl group in PLLA irradiated with higher laser fluences [22], there was a need to perform a biological test aimed at determining the cytotoxicity of the material. The performed biological assessment found no cytotoxic activity of the examined polymers, however, a mild cytotoxic effect was observed after laser treatment (F_2_ and F_3_). In accordance with the 10993-5 standard [29], changes in the culture over grade 2 are considered to be cytotoxic effect. Nevertheless, no cell colonization of the specimen surfaces was observed, which indicates that the examined surfaces of the materials do not have properties conducive to cell adhesion despite an improvement in the PLLA surface properties after irradiation with a CO_2_ laser, i.e., increased roughness and surface wettability.

The micromechanical properties of unmodified PLA have been very rarely tested using the instrumented indentation method [49,50] and, to the best of our knowledge, so far no one has tested surface-modified PLA. Microhardness and elastic modulus of neat and extruded PLA were tested using a diamond Berkovich indenter with indentation load of 0.5 mN, resulting in the maximum indentation depths of, respectively, 1.5 µm and [49] 1 µm [50]. The elastic modulus of extruded PLA ranged from 3.9 ± 0.47 GPa on specimen edges to 4.1 ± 0.26 GPa in the middle of the specimen, while modulus and hardness of neat PLA were uniform and equal to 4.6 ± 0.47 GPa and 0.23 ± 0.034 GPa, respectively [50]. For thin films of pure PLA, Ajala et al. [49] obtained lower modulus and hardness values of 0.95 and 0.03 GPa, respectively. Modulus and hardness of unmodified PLLA presented here are equal to 2.1 ± 0.9 GPa and 0.2 ± 0.05 GPa, respectively. The mechanical parameters of laser-irradiated PLA with F_1_ and F_2_ accumulated fluencies determined in instrumented indentation tests increased, thus reinforcing the surface. For the highest accumulated fluence, the determined parameters sharply decreased, resulting in significantly greater indentation depth (up to almost 2.5–3.5% of the total thickness of the specimen crossing the boundary of the surface layer). Laser irradiation of the PLLA surface with accumulated fluence of 71 J/cm^2^ led to a substantial weakness of both the surface and subsurface layers, with even lower parameters than those reported for non-modified PLA.

In the presented results, the mechanical parameters of the polymer were altered from stiff (groups F_1_ and F_2_) to compliant (F_3_) while simultaneously changing the surface topography and wettability. The two features with the strongest impact on cell viability are chemical interactions and surface topography. The possibility of controlled structuring of the PLLA surface and the observed effect of irradiation on the polymer properties allow the design of its properties, selectively and locally. This way it is possible to adjust the structure of the implant surface so as to ensure the most favorable conditions of cooperation with the surrounding tissues, thus contributing to the reduction in tissue regeneration time.

## Figures and Tables

**Table 1:** Process parameters for CO_2_ laser surface treatments of PLLA specimens.

Specimen	Optical Power *P* (W)	Scanning Speed *V* (cm/s)	Hatching (Line-to-Line Space) h (μm)	Pulse Repetition Rate (*PRR*) (Hz)	Pulse Energy *E_i_* (mJ)	Accumulated Fluence *F_A_* (J/cm^2^)
Reference	-	-	-	-	-	-
F_1_	0.447	7.1	25.4	2.8	15.5	24
F_2_	0.867				31.0	48
F_3_	1.286				45.8	71

**Table 2:** Roughness parameters describing surface topography of micro-patterned PLLA.

Roughness Parameters	Reference	F_1_	F_2_	F_3_
R_z_ $\left(\bar{X}\pm SD\right)$ (µm)	0.10 ± 0.04	0.21 ± 0.05	2.87 ± 0.31	6.67 ± 0.66
R_a_ * (µm)	0.02	0.04	0.57	1.33

* Values derived from the following correlation: R_a_ = 1/5 R_z_.

**Table 3:** Micromechanical properties presented as average and standard deviation ($\bar{X}\pm SD$) determined in the indentation test for references and irradiated specimens.

Specimen Type	E_IT_ (GPa)	H_IT_ (MPa)	HV	hm (μm)	We (nJ)	Wp (nJ)
Ref	2.1 ± 0.9	200.5 ± 53.2	18.9 ± 5.0	6.1 ± 0.9	116.0 ± 46.7	145.7 ± 27.3
F_1_	3.4 ± 0.8	300.2 ± 54.9	28.3 ± 5.2	4.7 ± 0.5	80.6 ± 13.0	113.4 ± 5.6
F_2_	4.1 ± 1.0	429.6 ± 205.8	40.5 ± 19.4	4.3 ± 0.7	73.6 ± 7.3	117.3 ± 9.3
F_3_	0.4 ± 0.03	115.9 ± 17.7	10.9 ± 1.7	1.2 ± 0.4	376.6 ± 40.2	166.7 ± 25.0

Legend: E_IT_—Young’s modulus, H_IT_—Instrumental microhardness, HV—Vickers microhardness, hm—Maximum indentation depth, We—Elastic strain energy, Wp—Plastic strain energy.
